# Towards Chemoenzymatic Syntheses of Both Enantiomers of Phosphoemeriamine

**DOI:** 10.3390/molecules29081799

**Published:** 2024-04-16

**Authors:** Piotr Kiełbasiński, Małgorzata Kwiatkowska, Piotr Łyżwa, Marian Mikołajczyk

**Affiliations:** Division of Organic Chemistry, Centre of Molecular and Macromolecular Studies, Polish Academy of Sciences, Sienkiewicza 112, 90-363 Łódź, Poland; malgorzata.kwiatkowska@cbmm.lodz.pl (M.K.); plyzwa@interia.pl (P.Ł.); marian.mikolajczyk@cbmm.lodz.pl (M.M.)

**Keywords:** chemoenzymatic synthesis, phosphoemeriamine, enzymatic kinetic resolution, chemical correlation, enantiomeric excess

## Abstract

An enzyme-promoted addition of nitromethane to the appropriate phosphorylated imine (aza-Henry reaction) intended to be used in the synthesis of the title phosphoemeriamine, a phospha-analog of emeriamine (aminocarnitine), failed due to the tautomerization of the imine to the corresponding enamine. Nevertheless, both enantiomers of phosphoemeriamine were synthesized in high yield and enantiomeric purity using another chemoenzymatic approach, starting with a crucial step involving a CAL-B-mediated acetylation of the appropriate racemic precursor—diethyl 2-amino-3-dimethylaminopropylphosphonate—under kinetic resolution conditions. The enzymatic reaction was very efficient and provided each enantiomeric product in acceptable yield and with enantiomeric excess of 91 and 92%. The following appropriate chemical transformations led to the desired enantiomers of phosphoemeriamine in the form of phosphoemeriamine sesquichloride with enantiomeric excess up to 90%.

## 1. Introduction

**Scheme 1:**
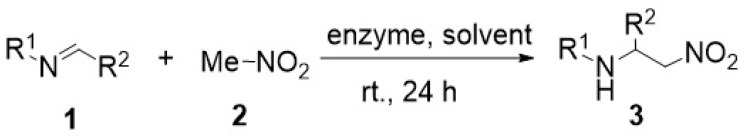
Enzyme-promoted aza-Henry reaction.

Continuing our interest in the enzyme catalytic promiscuity [1], i.e., the ability of a single active site of the enzyme to catalyze more than one reaction, particularly those which are different from that designed in studies published in Nature [2,3,4,5,6], we have recently reported on our investigations of the enzyme-promoted addition of nitromethane to aldimines (i.e., the aza-Henry reaction). We succeeded in obtaining, for the first time, the desired products of this type of addition **3** in the yields of 14–81%. The most efficient enzymes turned out to be lipase TL from *Pseudomonas stutzeri* and oxynitrilase from *Arabidopsis thaliana*. We have expected that the compounds **3** will be produced with high enantiomeric excess. However, much to our disappointment, all the reactions investigated turned out to be non-stereoselective (Scheme 1) [7].

Although the above results have not been fully satisfactory, we have decided to apply the newly elaborated procedure in the synthesis of a particular diaminophosphonic acid, namely phosphoemeriamine **8**, a phospha-analog of emeriamine (aminocarnitine). The latter is a natural amino acid showing interesting biological properties. It behaves as an inhibitor of fatty acid oxidation and acts as a hypoglycemic and antiketogenic agent [8,9,10,11]. It should be stressed that both enantiomers of phosphoemeriamine have so far been only synthesized ones, using an enantiomeric sulfinyl group as a chiral auxiliary. This methodology was developed in our department and has proven to be very efficient [8,9,10,11]. The synthetic strategy was based on a highly diastereoselective addition of the *O*,*O*-diethyl methylphosphonate carbanion to (*S*)-*N*-(*p*-toluenesulfinyl)cinnamaldimine, followed by isolation of the major diastereoisomeric β-amino adduct and its further conversion to the desired target through proper transformations. However, to obtain the opposite enantiomer, it would be necessary to apply the (*R*) enantiomer of the starting aldimine and to repeat the whole troublesome transformation procedures. To overcome these obstacles, we have decided to develop an environmentally friendly, chemoenzymatic methodology, which would use the approach shown above.

## 2. Results and Discussion

**Scheme 2:**
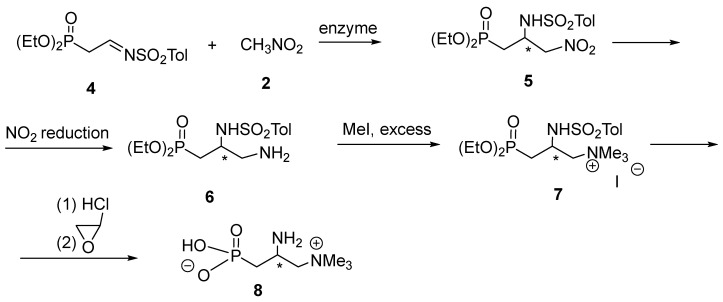
Envisaged synthesis of phosphoemeriamine **8** via the addition of nitromethane **2** to *N*-*p*-toluenesulfonyl-2-diethoxyphosphorylethanimine **4**. * denotes chirality centre.

Our plan to accomplish the synthesis of phosphoemeriamine based on the aza-Henry reaction is shown in Scheme 2. Thus, *N*-*p*-toluenesulfonyl-2-diethoxyphosphorylethanimine **4** was planned to be treated with nitromethane in the presence of various enzymes (which were reported efficient in the cited publication [7]).

**Scheme 3:**
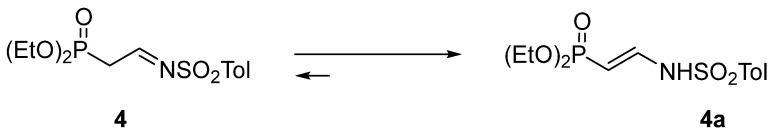
Tautomerization of *N*-*p*-toluenesulfonyl-2-diethoxyphosphorylethanimine **4**.

First, we attempted to synthesize substrate imine **4** via condensation of α-diethoxy phosphorylacetaldehyde with *p*-toluenesulfonamide, according to a procedure in the literature [12]. However, this derivative turned out to be useless in the envisaged transformation since it easily underwent tautomerization to the corresponding enamine **4a**, thus making the appropriate addition impossible (Scheme 3). The same transformation happened with the *N*-toluenesulfinyl analog of **4** since it behaved in a similar way.

In continuing our efforts, we decided to employ another chemoenzymatic approach. The latter is a recently and widely used application in asymmetric synthesis [13,14]. One of the crucial parts of this approach would comprise a kinetic resolution of an appropriate racemic precursor [15,16]. Noteworthy, a similar approach was successfully applied in our laboratory earlier in the synthesis of enantiomers of phosphocarnitine [17].

**Scheme 4:**
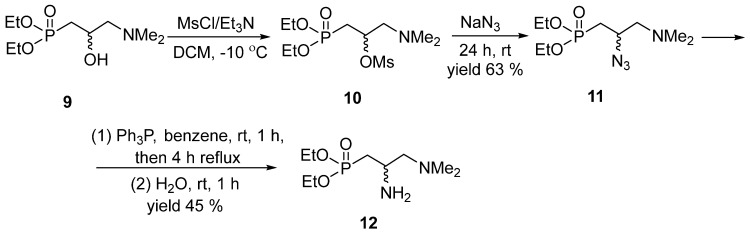
Synthesis of substrate **12** for a kinetic resolution.

Thus, we chose racemic diethyl 2-amino-3-*N*,*N*-dimethylaminopropylphosphonate **12** as a substrate in the lipase-promoted acetylation under kinetic resolution conditions. It was synthesized by us starting from diethyl 2-hydroxy-3-*N*,*N*-dimethylaminopropylphosphonate **9 [18]**. The ensuing mesylation resulted in **10** and followed by its reaction with sodium azide gave diethyl 2-azido-3-*N*,*N*-dimethylaminopropylphosphonate **11**. The latter was subjected to the Staudinger reaction with triphenylphosphine to furnish the desired racemic substrate **12** (Scheme 4).

**Scheme 5:**
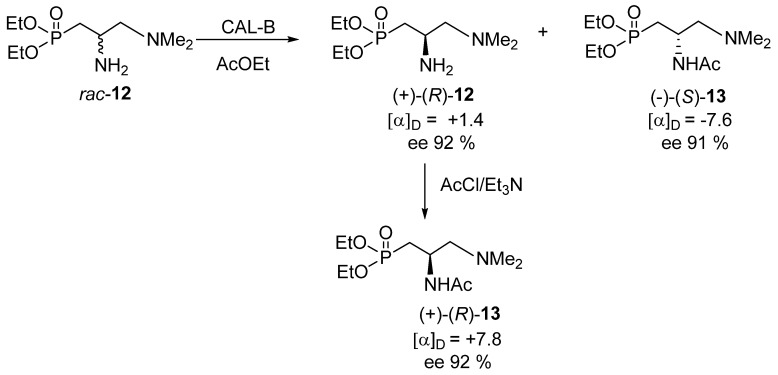
Enzymatic kinetic resolution of *rac*-**12**.

The kinetic resolution of *rac*-**12** was achieved via its *N*-acetylation with ethyl acetate in the presence of various hydrolytic enzymes [e.g., CAL-B (Novozym 435)—lipase acrylic resin from *Candida antarctica*, PFL—lipase from *Pseudomonas fluorescens*, TL—lipase from *Pseudomonas stutzeri* and others], of which lipase from *Candida antarctica*, CAL-B (Novozym 435) proved most efficient (Scheme 5) [19,20,21].

This approach turned out to be successful and both the recovered substrate—amine (+)-**12** and the acetylation product—*N*-(diethoxyphosphoryl)methyl-(*N′*,*N′*-dimethyl-aminomethyl)acetamide (−)-**13** were obtained in 30% and 42% yield, respectively, and with enantiomeric excess up to 92% (e.r. 96:4) and 91% (e.r. 95.5:4.5), respectively. Their absolute configurations were not determined at this stage (vide infra), but the recovered substrate (+)-**12** was chemically acetylated to give (+)-**13** exhibiting almost the same value (though obviously of the opposite sign) of optical rotation as (−)-**13**, which could be taken as proof of the efficiency of the kinetic resolution (Scheme 5). The enantiomeric excess of the recovered amine (+)-**12** was determined by ^31^P NMR using optically pure (+)-(*R*)-*t*-butylphenylphosphinothioic acid as a chiral solvating agent [22,23]. In turn, the enantiomeric excess of the acetylation product, acetamide **13**, was determined on the basis of its optical rotation and via a chemical correlation shown in Scheme 5.

**Scheme 6:**
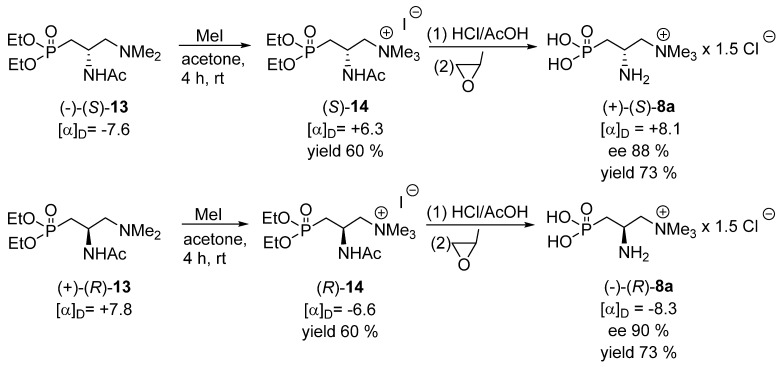
Final synthesis of each enantiomer of phosphoemeriamine sesquichloride **8a**.

The ensuing chemical transformations of each enantiomer of the acetamide **13** were as follows (Scheme 6). The subsequent methylation of the terminal tertiary amino group furnished the final phosphoemeriamine precursor, trimethyl (2-acetamido)-(3-diethoxyphosphoryl)-propylammonium iodide **14**. It was subjected to hydrolysis, using the procedure described in a previous publication [11] to give the desired products **8**. It must be stressed that the enantiomers of **8** were obtained in the form of the complexes in which two molecules of phosphoemeriamine were complexed with one additional chloride anion, indicated here as **8a**, which is in accordance with the result described earlier [11]. Hence, in one molecule of the product, prepared as above, the ratio between the phosphoemeriamine cation and the chloride anion is 1:1.5; thus, the final structure may be described as phosphoemeriamine sesquichloride **8a**. In this way, each enantiomer of phosphoemeriamine sesquichloride **8a** was obtained and characterized and their absolute configurations were ascribed by comparing the sign of their optical rotation with that described previously (for the (−)-(*R*) enantiomer of >99% ee, [α]_D_ = − 9.2) [11], although a partial racemization was observed in our work, most probably at the stage of the acidic hydrolysis of **14**. The determination of the absolute configuration of the final products enabled us to also determine absolute configurations of the intermediates **12**, **13** and **14** since their transformations into phosphoemeriamine sesquichloride proceeded without involvement of the stereogenic center (Scheme 5 and Scheme 6).

## 3. Experimental Section

### 3.1. General Information

All solvents were dried and distilled prior to use. The starting materials and enzymes were purchased from Merck (Poznan, Poland), Sigma-Aldrich (Poznań Poland, TCI Chemicals (Trimen, Łódź, Poland) or Fluorochem (Hadfield, UK). The synthesized products were purified by column chromatography on a Merck 60 silica gel (0.063–0.200 mm) or preparative plate chromatography using a Merck 60 F_254_ silica gel plate (2.5 mm). TLC was performed on a Merck 60 F_254_ silica gel plate (0.25 mm). All the reactions were run in duplicate. The NMR spectra were recorded in CDCl_3_ or D_2_O using a Bruker AV III 500 spectrometer (Poznań, Poland) at 500 MHz (^1^H), 126 MHz (^13^C) and 202 MHz (^31^P). Mass spectra, including HRMS, were measured on a Finnigan MAT instrument (Bremen, Germany) Optical rotations were measured on a Perkin-Elmer 241MC polarimeter (Uberlingen, Germany).

### 3.2. Diethyl 2-Azido-3-N,N-dimethyloaminopropylphosphonate ***11***

To the solution of diethyl 2-hydroxy-3-*N*,*N*-dimethyloaminopropylposphonate **9** (1 g, 4.16 mmol), in dry methylene chloride (5 mL) was added at −10 °C along with triethylamine (0.7 mL) and mesyl chloride (0.518 g, 350 μL, 4.4 mmol). The reaction mixture was stirred at this temperature for 1 h and, without separation of the mesyl derivative **10,** sodium azide (0.546 g, 8.2 mmol) was added. After stirring for 24 h at room temperature, water (5 mL) was added and the organic fraction was separated and dried over MgSO_4_. The crude reaction mixture was purified using column chromatography (silica gel, CH_2_Cl_2_:MeOH 20:1) to give **11**. Yield 0.7 g, 63%.

^1^H NMR (CDCl_3_): δ = 1.29 (t, *J* = 7 Hz, 6H, CH_3_CH_2_); 1.38 ÷ 1.73 (m, 1H, CH_2_P); 1.83 ÷ 2.03 (m, 1H, CH_2_P); 2.23 (s, 6H, NMe_2_); 3.02 ÷ 3.18 (m, 1H, CHNMe_2_); 3.29 ÷ 3.48 (m, 2H, CH_2_N); 3.99 ÷ 4.13 (q, *J* = 7 Hz, 4H, CH_3_CH_2_).
^31^P NMR (CDCl_3_): δ = 30.1.
HRMS (FAB) calcd for C_9_H_22_P_1_N_4_O_3_ (M + 1); 265.1429 found 265.1430.

### 3.3. Diethyl 2-Amino-3-N,N-dimethyloaminopropylphosphonate Rac-***12***

To the azide **11** (0.3 g, 1.36 mmol) dissolved, benzene (5 mL) was added triphenylphosphine (0.325 g, 1.24 mmol) and the solution was stirred for 1 h at room temperature, then for 4 h at reflux. After cooling to room temperature, water (5 mL) was added, stirred intensively for 1 h and the water fraction separated (it was repeated 3 times). The water fractions were collected and evaporated, the residue formed was dissolved in methylene chloride (5 mL) and dried over MgSO_4_. The crude product was purified using column chromatography (silica gel, CH_2_Cl_2_: MeOH 10:1, then MeOH). Yield 0.122 g, 45%.

^1^H NMR (CDCl_3_): δ = 1.29 (t, *J* = 7 Hz, 6H, CH_3_CH_2_); 1.38 ÷ 1.55 (m, 1H, PCH); 1.76 ÷ 2.00 (m, 2H, CHP, NH_2_); 2.16 (s, 6H, N(CH_3_)_2_; 2.54 ÷ 2.60 (m, 1H, CHNH_2_); 2.78 ÷ 2.99 (m, 2H, CH_2_N); (q, 4H, CH_3_CH_2_).
^31^P NMR (CDCl_3_): δ = 31.7.
^13^C NMR (CDCl_3_): δ = 16.4, 21.1 (d, *J* = 137.7 Hz, PCH_2_); 39.6; 43.4; 61.4.
HRMS (FAB) calcd for C_9_H_24_P_1_N_2_O_3_ (M + 1); 239.1525 found 239.15344.

### 3.4. N-(Diethoxyphosphoryl)methyl-(N′,N′-dimethylaminomethyl)acetamide (+)–(R)-***13***

To the solution of an enantiomerically enriched diethyl 2-amino-3-*N*,*N*-dimethyloaminopropylphosphonate (+)-(*R*)-**12** (0.1 g, 0.42 mmol) and triethylamine (0.043 g, 0.42 mmol) in THF (10 mL) at −78 °C and under nitrogen, acetyl chloride (0.033 g, 0.42 mmol) was added dropwise. The reaction mixture was stirred at this temperature for 4 h. After warming to room temperature, water (5 mL) was added, product was extracted with CH_2_Cl_2_ (3 × 5 mL) and dried over MgSO_4_, to give (+)-(*R*)-**13**. Yield 0.095 g, 80%, [α]_D_ = −7.8, c = 1, CH_2_Cl_2_.

^1^H NMR (CDCl_3_): δ = 1.31 (t, *J* = 7 Hz, 6H, CH_3_CH_2_); 1.40 ÷ 1.64 (m, 1H, CHP); 1.90 ÷ 2.10 (m, 1H, CHP); 1.95 (s, 3H, CH_3_O); 2.20 (s, 6H, NCH_3_)_2_); 2.83 ÷ 3.31 (m, 2H, CH_2_); 3.56 ÷ 3.70 (m, 1H, CH); 4.01 ÷ 4.15 (m, 4H, CH_3_CH_2_); 6.53 (bs, 1H, NHCO).
^31^P NMR (CDCl_3_): δ = 30.9.
^13^C NMR (CDCl_3_): δ = 16.3, 22.5 (d, *J* = 138.15 Hz, PCH_2_); 23.0; 39.6; 40.9; 52.7; 57.3; 61.8; 169.9 (CO).
HRMS (FAB): calcd for C_11_H_25_P_1_N_2_O_4_ (M + 1); 281.1628 found 281.1630.

### 3.5. Kinetic Enzymatic Resolution of Diethyl 2-Amino-3-N,N-dimethylaminopropyl-phosphonate Rac-***12***

To the aminophosphonate rac-**12** (35 mg, 1.47 mmol) in CH_2_Cl_2_ (6 mL), ethyl acetate (3.5 mL) and CAL-B (70 mg) were added and the suspension was warmed to 35 °C and vigorously stirred. The reaction progress was controlled using the ^31^P NMR technique. After the conversion reached 50%, the enzyme was filtered off and the solvent evaporated. The crude reaction mixture was separated using flash chromatography (silica gel, CH_2_Cl_2_:MeOH 5:1, then MeOH) to give 17.3 mg (42.1%) of (−)-**13**, [α]_D_ = −7.6, c = 1, CH_2_Cl_2_ and 10.1 mg (30%) of (+)-**12**, [α]_D_ = +1.4, c = 1, CH_2_Cl_2_ (both yield values with respect to the 50% yields of the substrate and the product, which can be produced in the enzymatic kinetic resolution).

### 3.6. Trimethyl-(3-diethoxyphosphoryl-2-acetamido)propylammonium Iodide ***14*** (S or R)

To a solution of **13** (0.1 g, 0.36 mmol) prepared as above in acetone (5 mL), methyl iodide (0.071 g, 0.5 mmol) was added dropwise under nitrogen. The reaction mixture was stirred at room temperature for 4 h. Acetone was evaporated, water (5 mL) added and then extracted with CH_2_Cl_2_ (3 × 2 mL). The water phase was evaporated and the oily residue was dissolved in CH_2_Cl_2_ (5 mL). The organic solution was dried over MgSO_4_ and evaporated to afford the crude product (a light brown solid), which upon column chromatography (CH_2_Cl_2_:MeOH 20:1), resulted in the desired iodide **14** as a yellowish solid. Yield 0.063 g, 60%. (+)-(*S*)-**14**: [α]_D_ = +6.3, c = 1, CH_2_Cl_2_; (−)-(*R*)-**14**: [α]_D_ = −6.6, c = 1, CH_2_Cl_2_.

^1^H NMR (D_2_O): δ = 1.27 (t, *J* = 7 Hz, 6H, CH_3_CH_2_); 1.96 (s, 3H, CH_3_CO); 2.60 ÷ 2.80 (m, 2H, PCH_2_); 3.11 (s, 9H, NCH_3_); 3.58 ÷ 3.82 (m, 3H, CH_2_CH); 4.13 (q, *J* = 7 Hz, 4H, CH_3_CH_2_).
^31^P NMR (D_2_O): δ = 26.7.
^13^C NMR (D_2_O): δ = 15.6; 22.0; 33.9 (d, *J* = 143.1 Hz); 39.0; 51.7; 64.2; 68.0; 174.7 (C=O).
HRMS (FAB) calcd for C_12_H_28_P_1_N_2_O_4_ 295.17924 found 295.1787.

### 3.7. 2-Amino-3-phosphoryl-1-trimethylammonium (Emeriamine) Sesquichloride ***8a*** (R or S)

Ammonium iodide **14** (0.063 g, 0.21 mmol) was refluxed with conc. hydrochloric acid (3 mL) and acetic acid (1 mL) for 5 h. Then, water (15 mL) was added and the aqueous solution was extracted with CH_2_Cl_2_ (4 × 3 mL). The water phase was evaporated to give a light-yellow oil. It was dissolved in EtOH (2 mL) and neutralized with propylene oxide. The white solid that had formed was filtered off, washed with EtOH (2 × 1 mL) and Et_2_O (3 mL), and dried over P_2_O_5_, affording the title product **8** in the form of white crystals. Yield 0.0287g, 73%. (+)-(*S*)-**8a**: [α]_D_ = +8.1, c = 1, CH_2_Cl_2_; (−)-(*R*)-**8a**: [α]_D_ = −8.3, c = 1, CH_2_Cl_2_.

^1^H NMR (D_2_O): δ = 4.14 ÷ 4.00 (m, 1H, CHNH_2_), 3.90 ÷ 3.66 (m, 2H, CH_2_), 3.18 (s, 9H, (CH_3_)_3_N), 2.11 ÷ 1.97 (m, 2H, CH_2_P).
^31^P NMR (D_2_O): δ = 16.5.
^13^C NMR (D_2_O): δ = 66.4, 52.3, 41.9, 29.9 (*d*, *J_PC_* = 129.8 Hz, PCH_2_).
HRMS: calcd for C_6_H_18_N_2_PO_2_ [M+] 197.1051 found 197.1055.
Elemental analysis for C_6_H_18_Cl_1.5_N_2_O_3_P (250.37): calcd for C 28.81, H 7.26, N 11.21; found C 29.30, H 7.71, N 10.85.

## 4. Conclusions

An attempt to synthesize enantiomeric phosphoemeriamine via the enzyme-promoted addition of nitromethane to aldimines (aza-Henry reaction) failed due to the tautomerization of the appropriate phosphorylated imine to the corresponding enamine. However, another chemoenzymatic approach, in which the crucial step involved a CAL-B-mediated acetylation of the appropriate racemic precursor—diethyl 2-amino-3-dimethylaminopropylphosphonate under kinetic resolution conditions—proved to be very efficient and provided each enantiomeric product (the starting amine and the acetamide formed) in acceptable yield and with enantiomeric excesses up to 92%. The following appropriate chemical transformations led to the desired enantiomers of phosphoemeriamine in the form of their sesquichlorides with ee up to 90%.

## Data Availability

Data are contained within the article and Supplementary Materials.

## Supplementary Materials

The following supporting information can be downloaded at: https://www.mdpi.com/1420-3049/29/8/1799/s1, ^31^P, ^1^H, ^13^C NMR spectra of new compounds.
