# Supplementary material for: Towards Chemoenzymatic Syntheses of Both Enantiomers of Phosphoemeriamine
Source: Molecules. 2024 Apr 16;29(8):1799. doi: 10.3390/molecules29081799 (PMC11154567; doi:10.3390/molecules29081799)

SUPPORTING INFORMATION

Towards Chemoenzymatic Syntheses of Both Enantiomers of Phosphoemeriamine

Piotr Kiełbasiński *, Małgorzata Kwiatkowska, Piotr Łyżwa and Marian Mikołajczyk

Division of Organic Chemistry, Centre of Molecular and Macromolecular Studies, Polish Academy of Sciences, Sienkiewicza 112, 90-363 Łódź, Poland;
malgorzata.kwiatkowska@cbmm.lodz.pl (M.K.); plyzwa@interia.pl (P.Ł.);
marian.mikolajczyk@cbmm.lodz.pl (M.M.)
* Correspondence: piotr.kielbasinski@cbmm.lodz.pl

Diethyl 2-azido-3-N,N-dimethyloaminopropylphosphonate **11**

^{31}P NMR ($CDCl_3$)

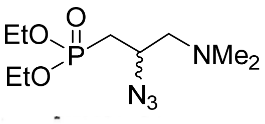

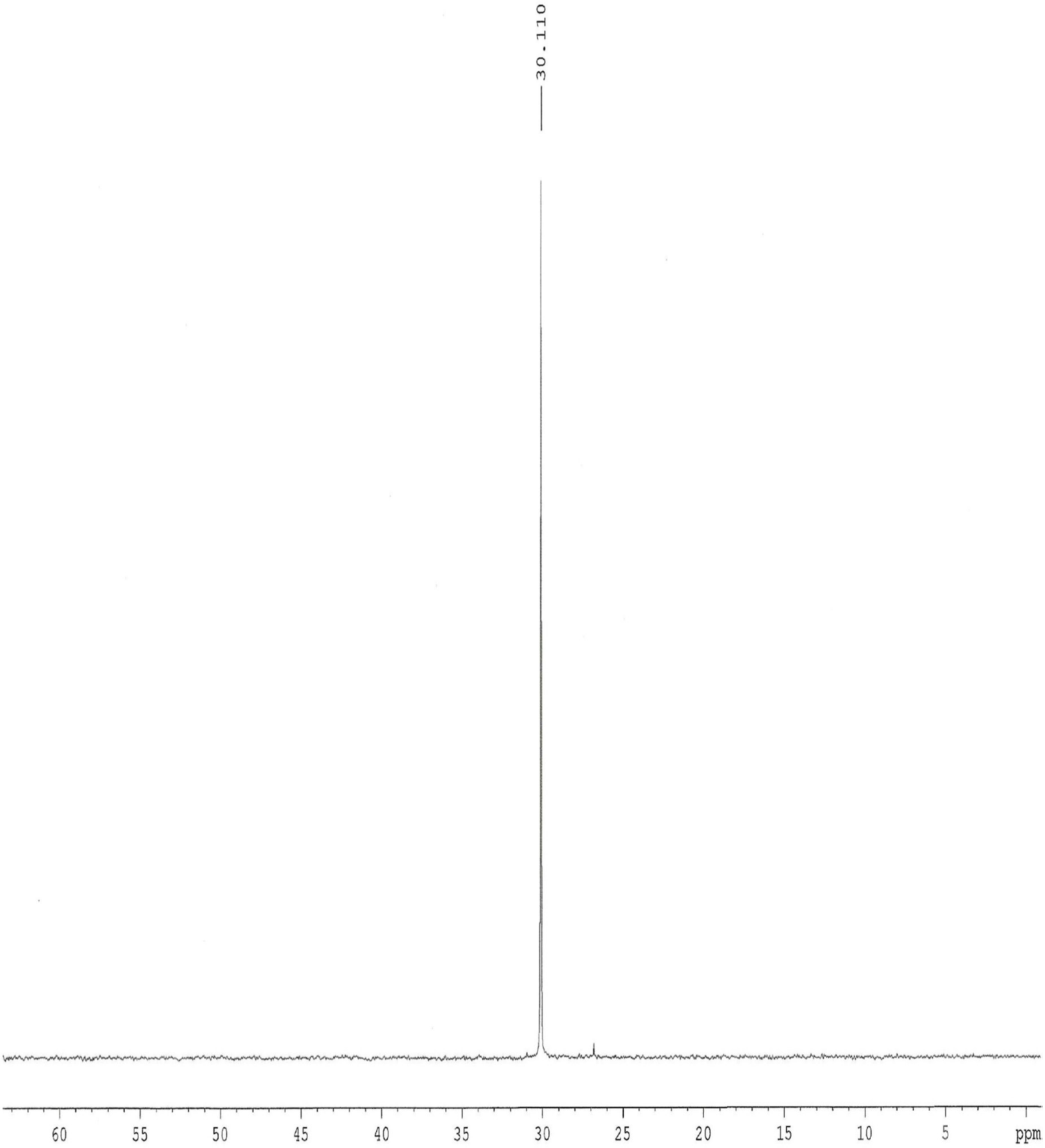

Diethyl 2-azido-3-N,N-dimethyloaminopropylphosphonate **11**

^{1}H NMR ($CDCl_3$)

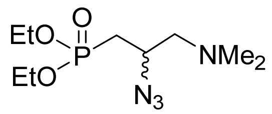

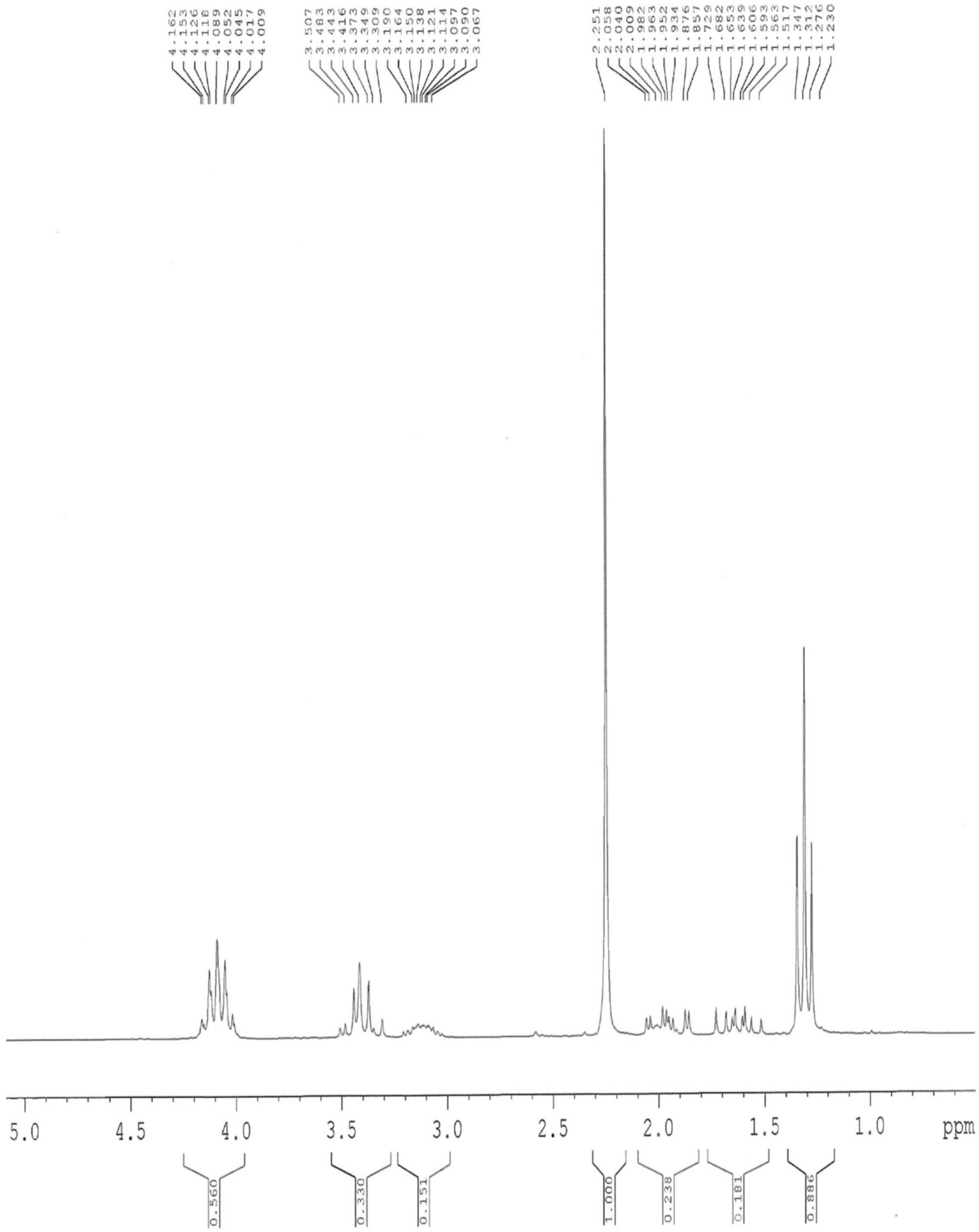

Diethyl 2-amino-3-N,N-dimethyloaminopropylphosphonate **12**
^{31}P NMR

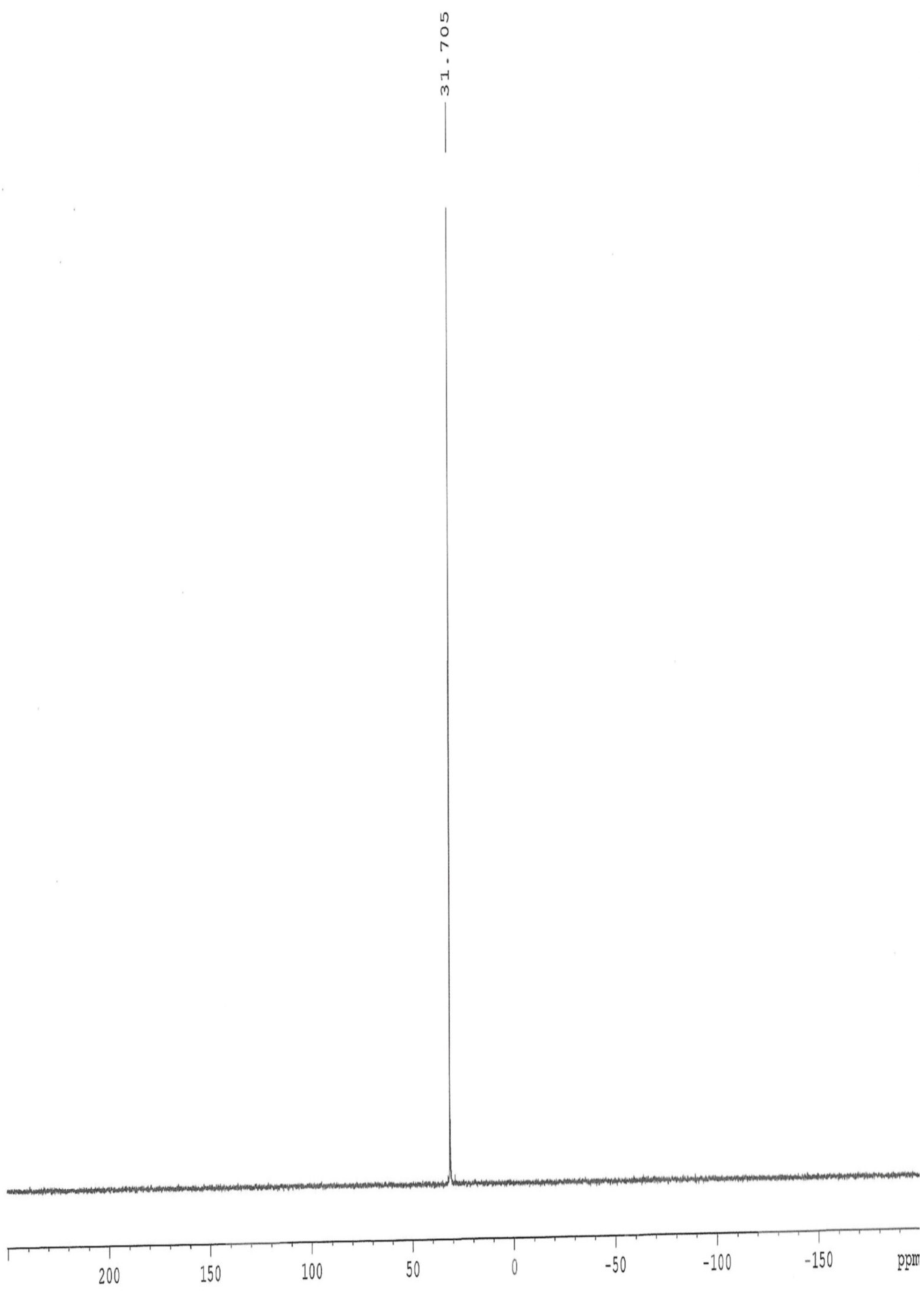

Diethyl 2-amino-3-N,N-dimethyloaminopropylphosphonate **12**

^{1}H NMR

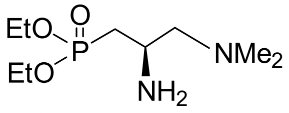

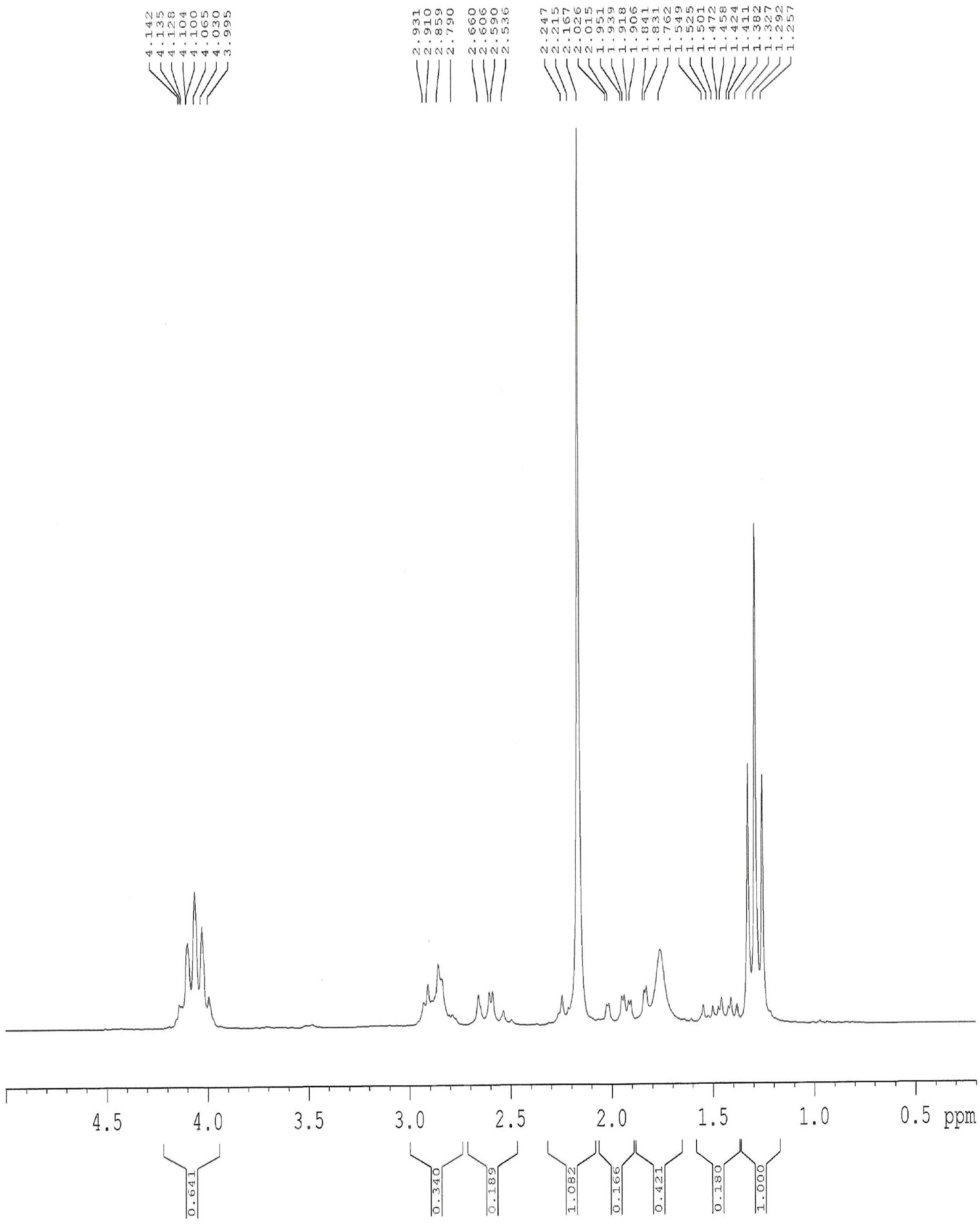

Diethyl 2-amino-3-N,N-dimethyloaminopropylphosphonate **12**

$^{13}C\{^{1}H\}$ NMR

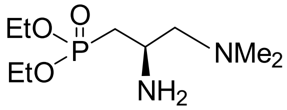

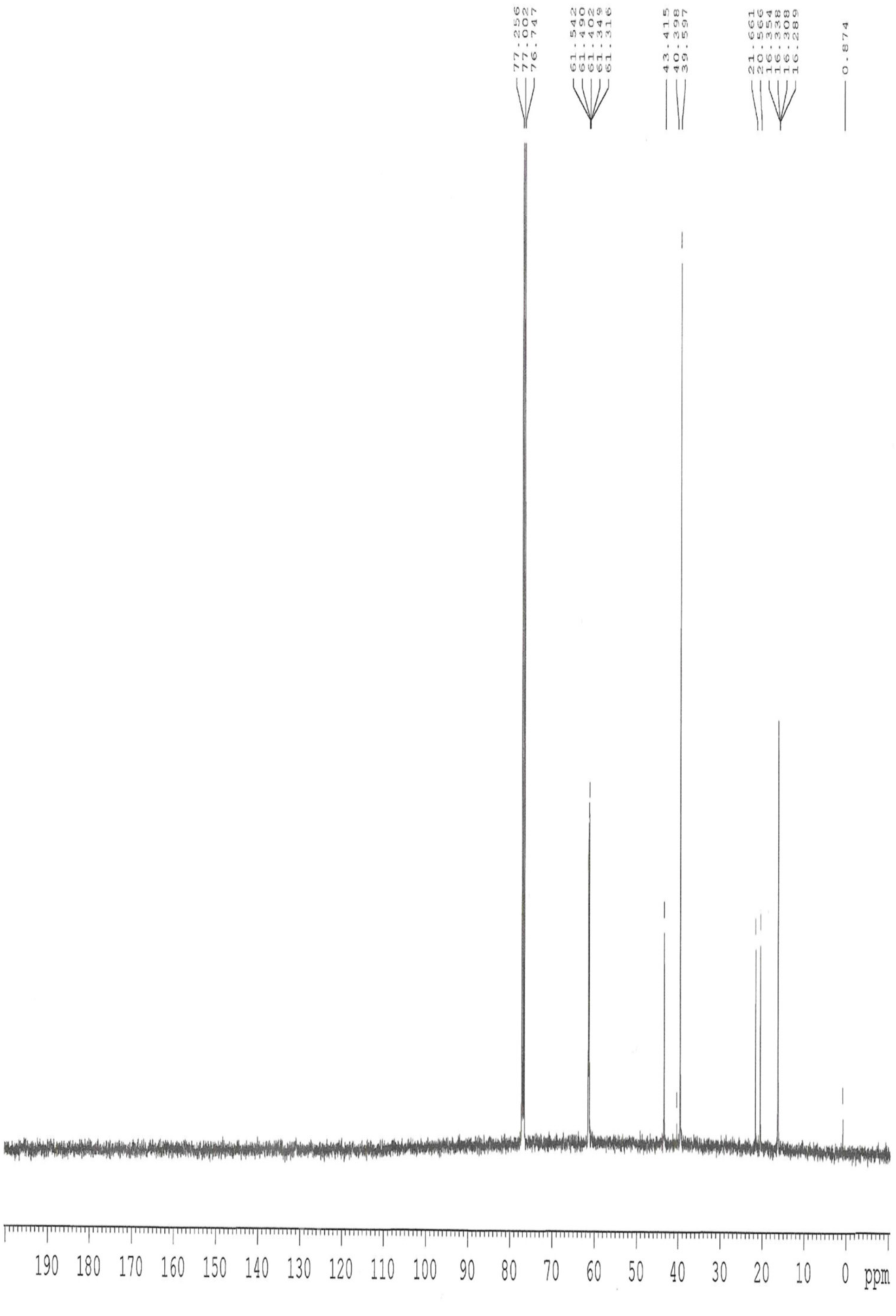

N-(Diethoxyphosphoryl)methyl(N',N'-dimethylaminomethyl)acetamide **13**

^{31}P NMR

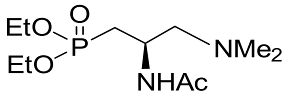

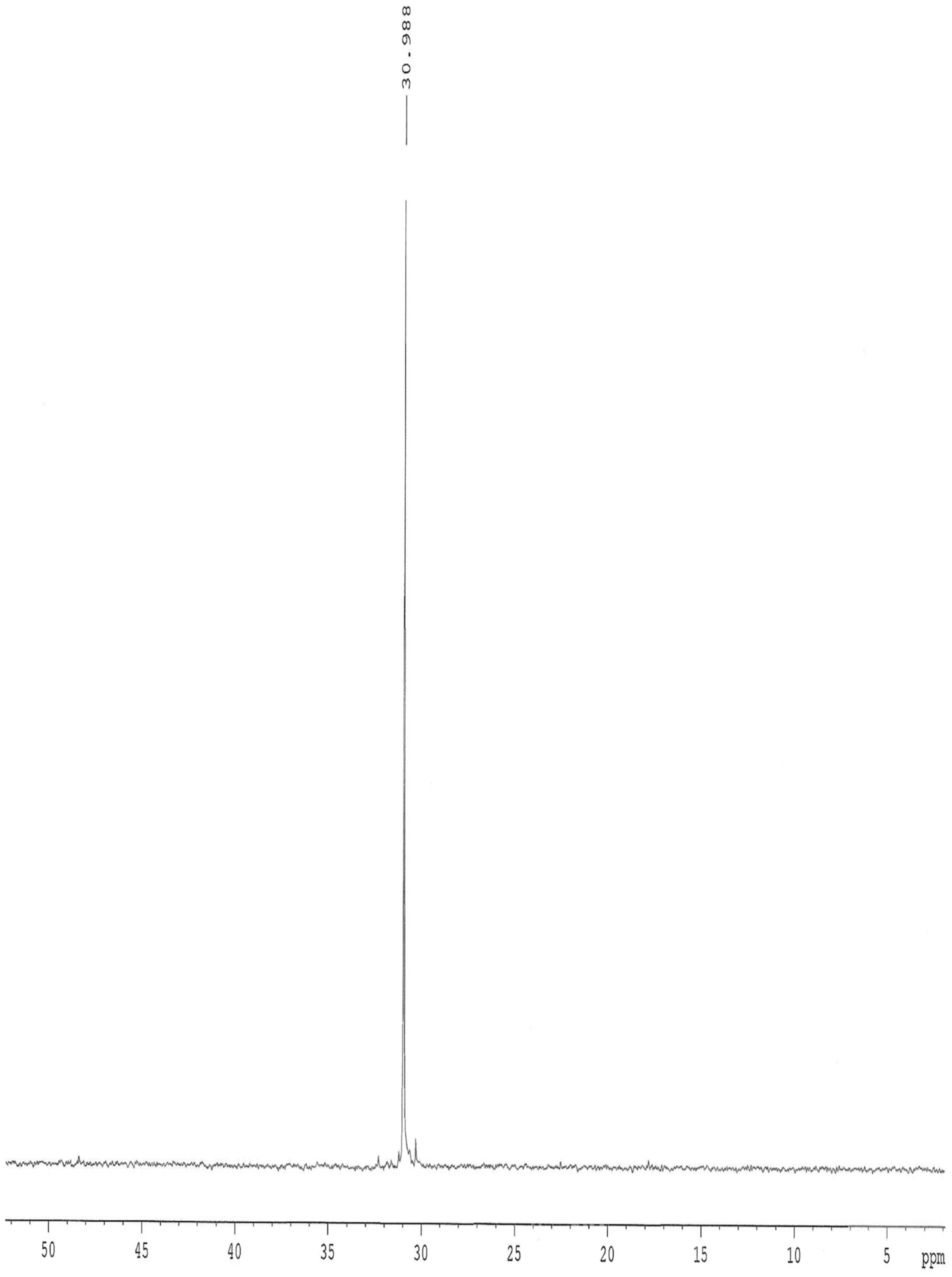

N-(Diethoxyphosphoryl)methyl(N',N'-dimethylaminomethyl)acetamide **13**

^{1}H NMR

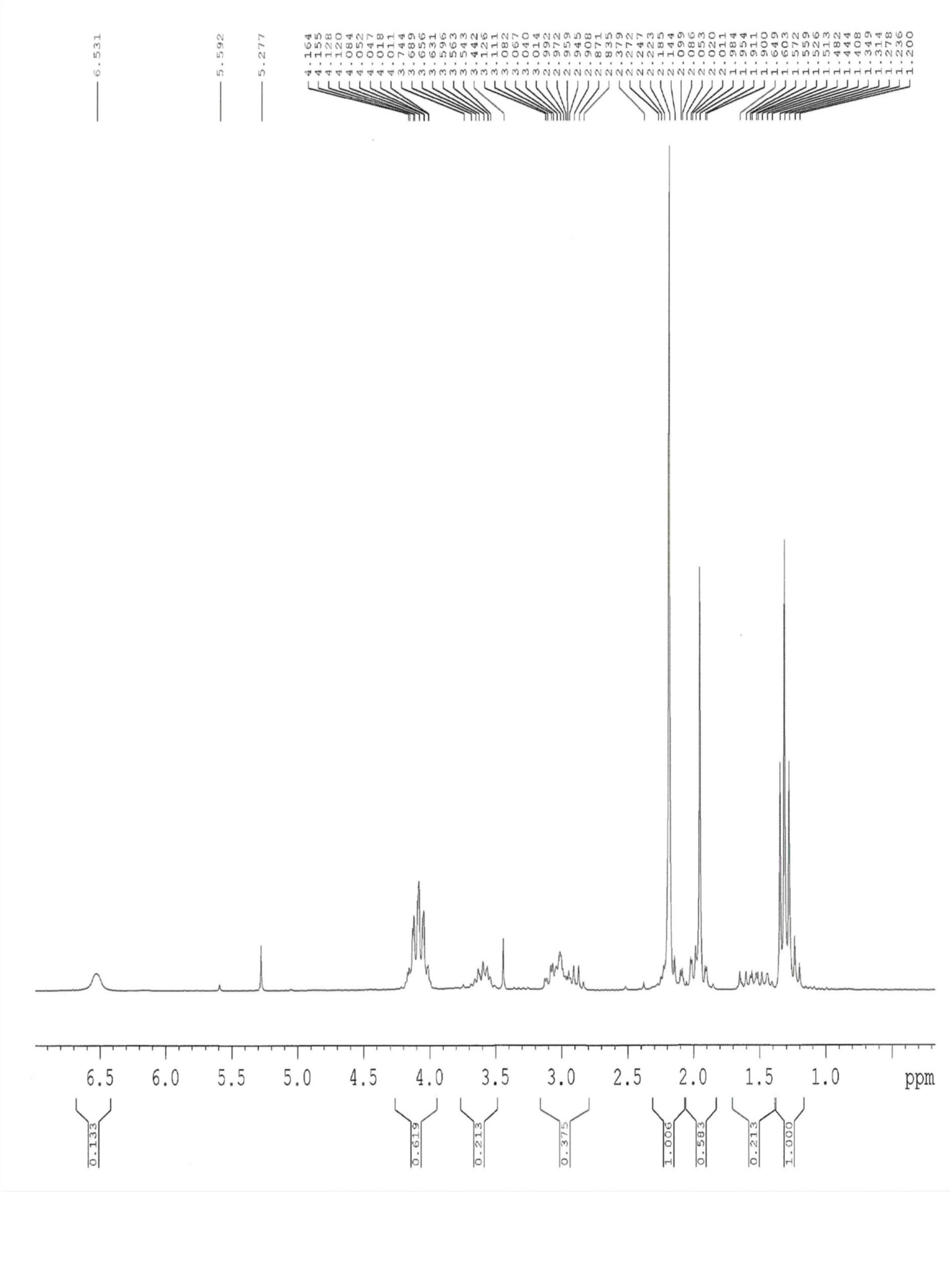

N-(Diethoxyphosphoryl)methyl(N',N'-dimethylaminomethyl)acetamide **13**

$^{13}C\{^{1}H\}$ NMR

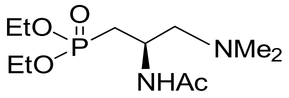

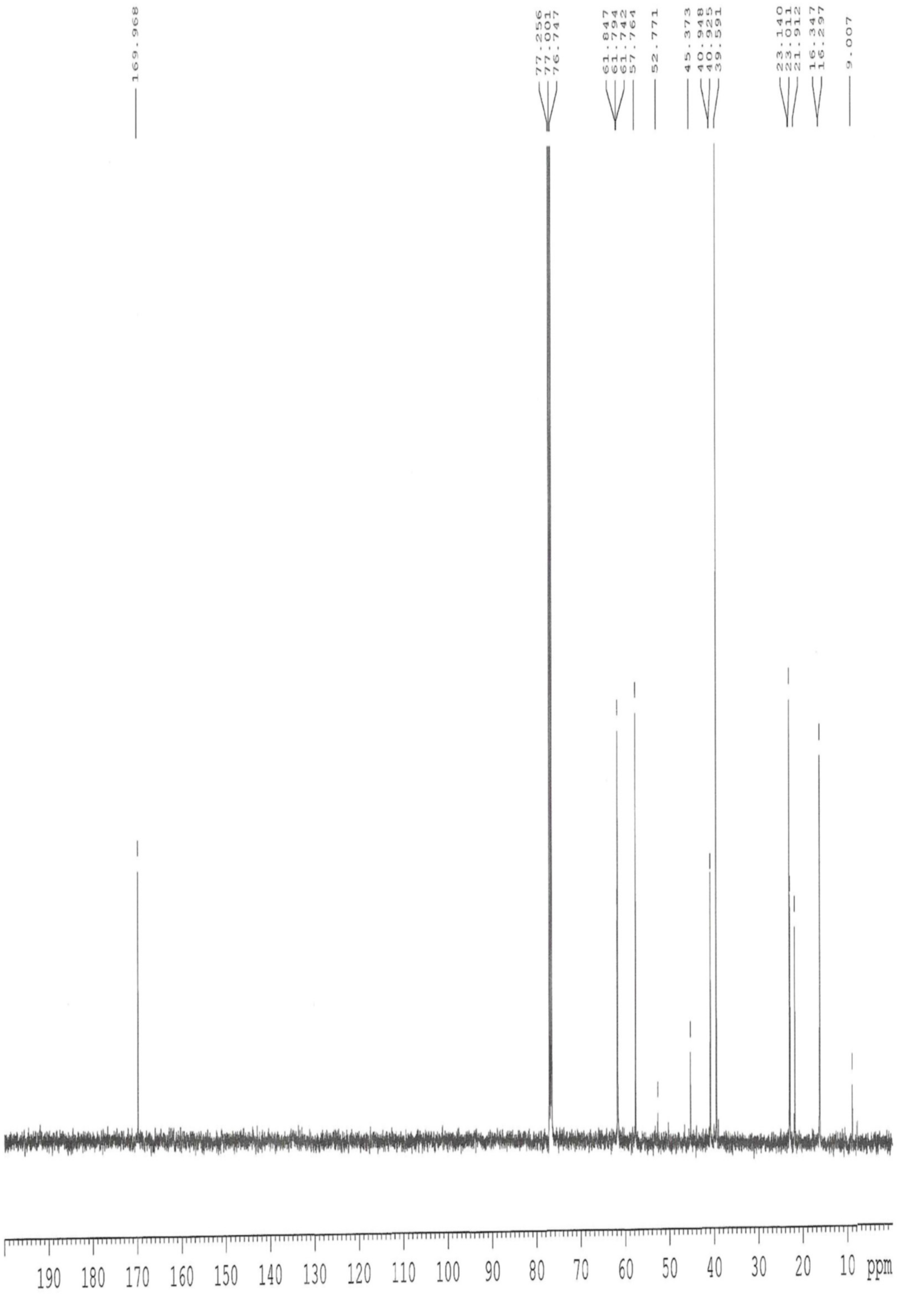

Trimethyl (2-acetamido)-(3-diethoxyphosphoryl)propylammonium iodide **14**

^{31}P NMR (D_2O)

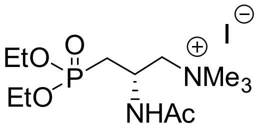

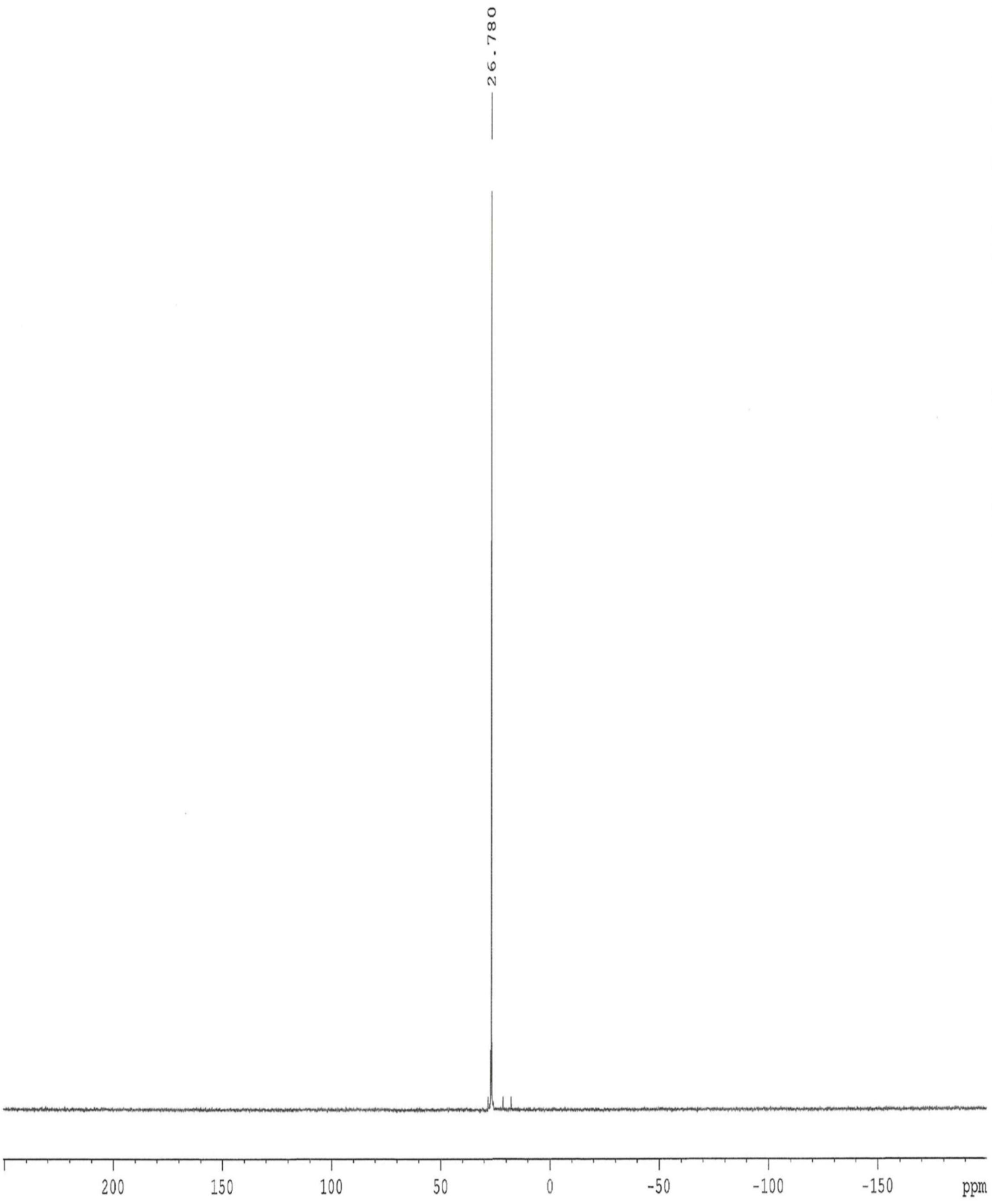

Trimethyl (2-acetamido)-(3-diethoxyphosphoryl)propylammonium iodide **14**

^{1}H NMR (D_2O)

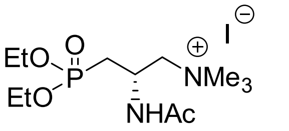

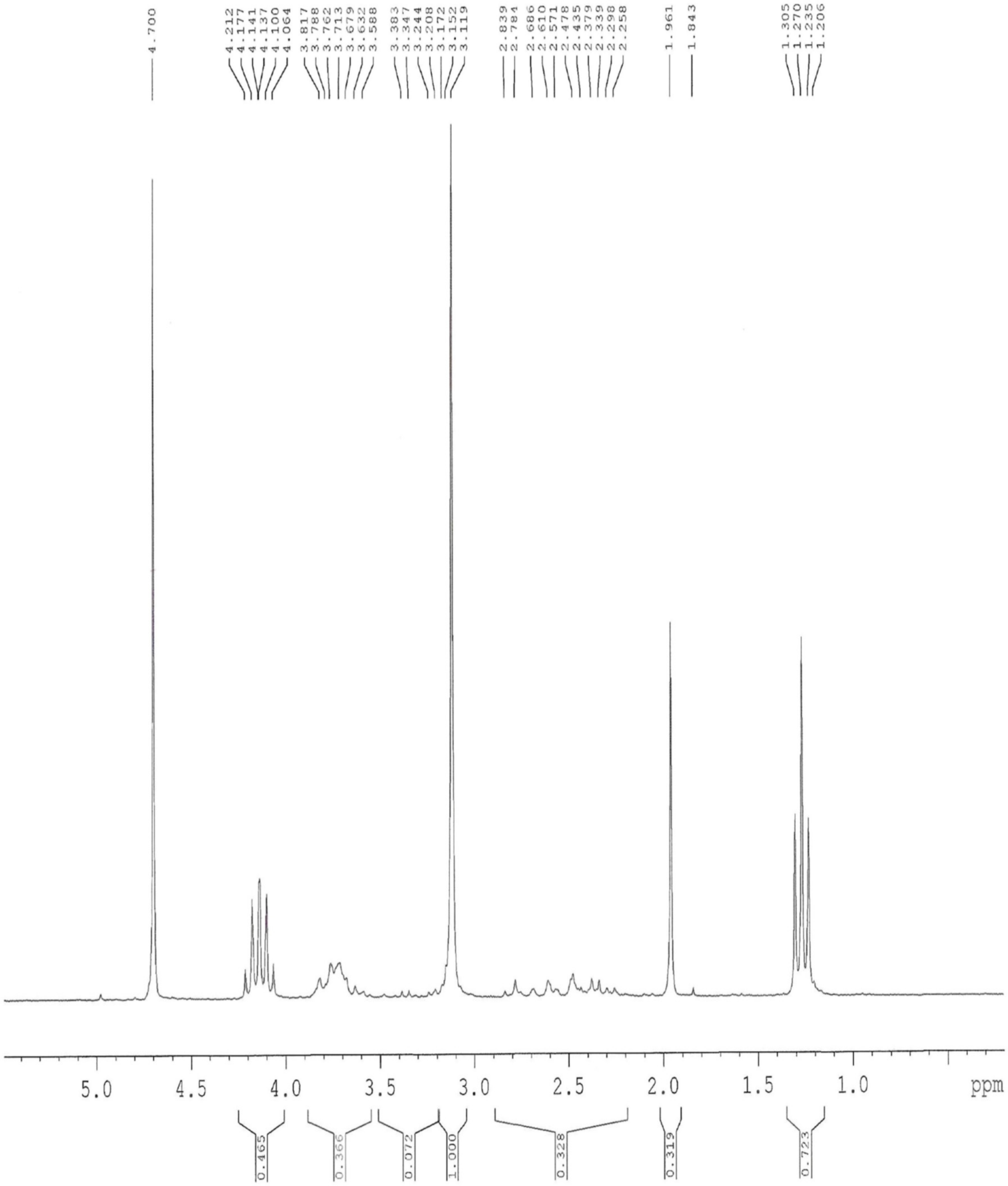

Trimethyl (2-acetamido)-(3-diethoxyphosphoryl)propylammonium iodide **14**

^{13}C{^{1}H} NMR (D_2O)

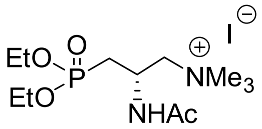

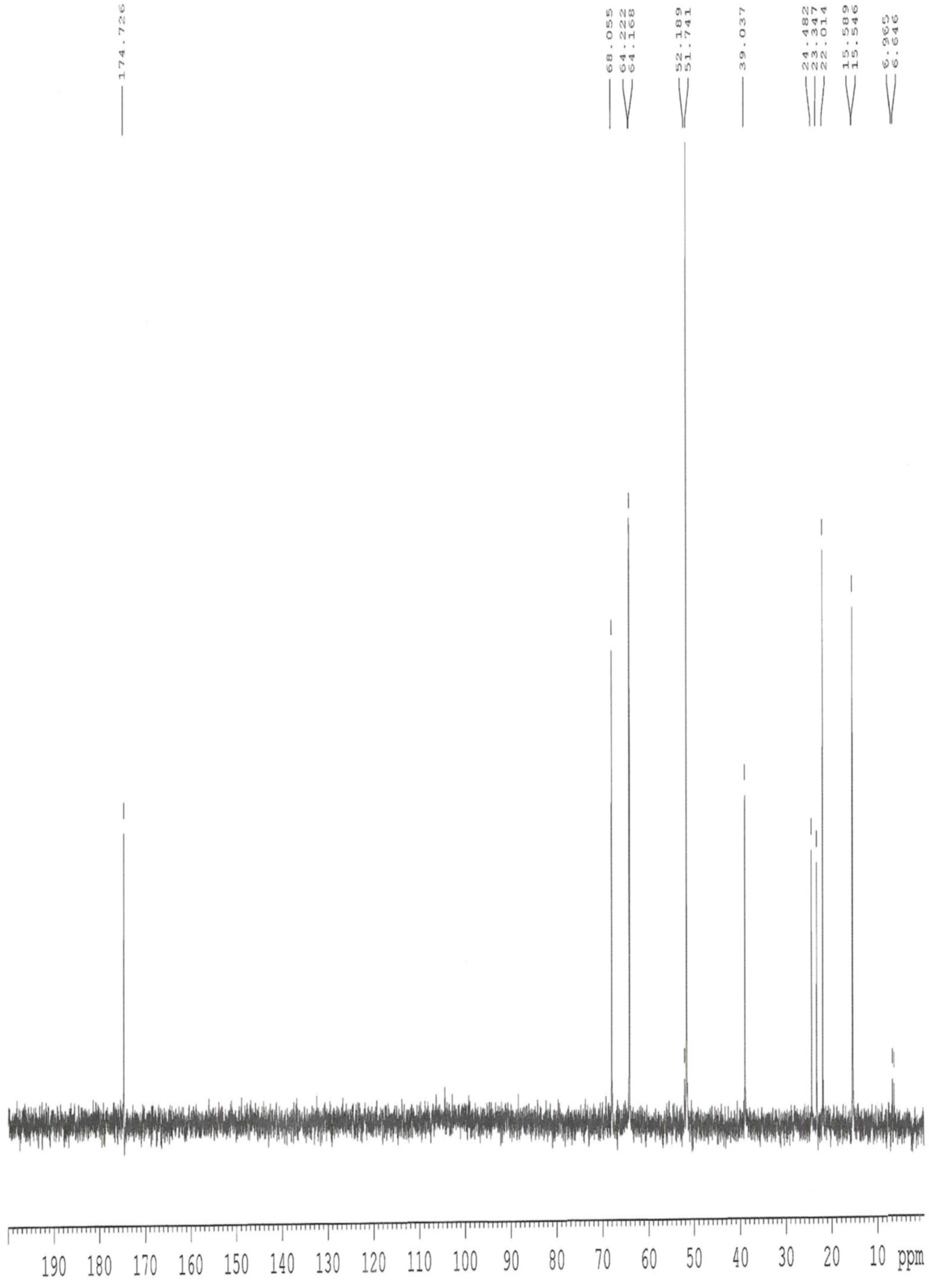

2-Amino-3-phosphoryl-1-trimethylammonium (emeriamine) sesquichloride ***8a***

^{31}P NMR (D_2O)

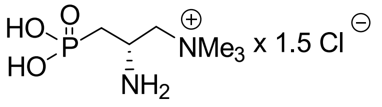

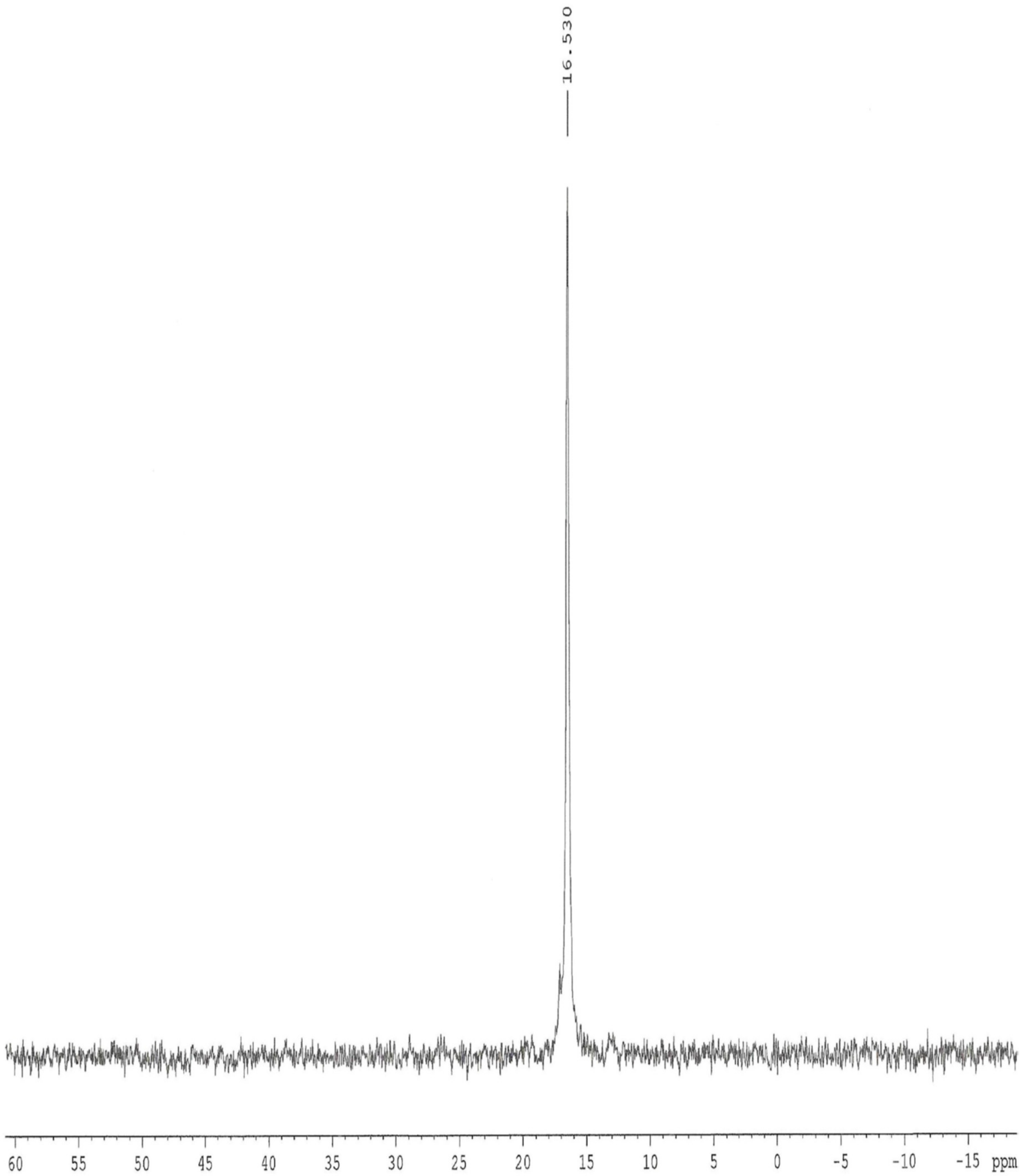

2-Amino-3-phosphoryl-1-trimethylammonium (emeriamine) sesquichloride ***8a***

^{1}H NMR (D_2O)

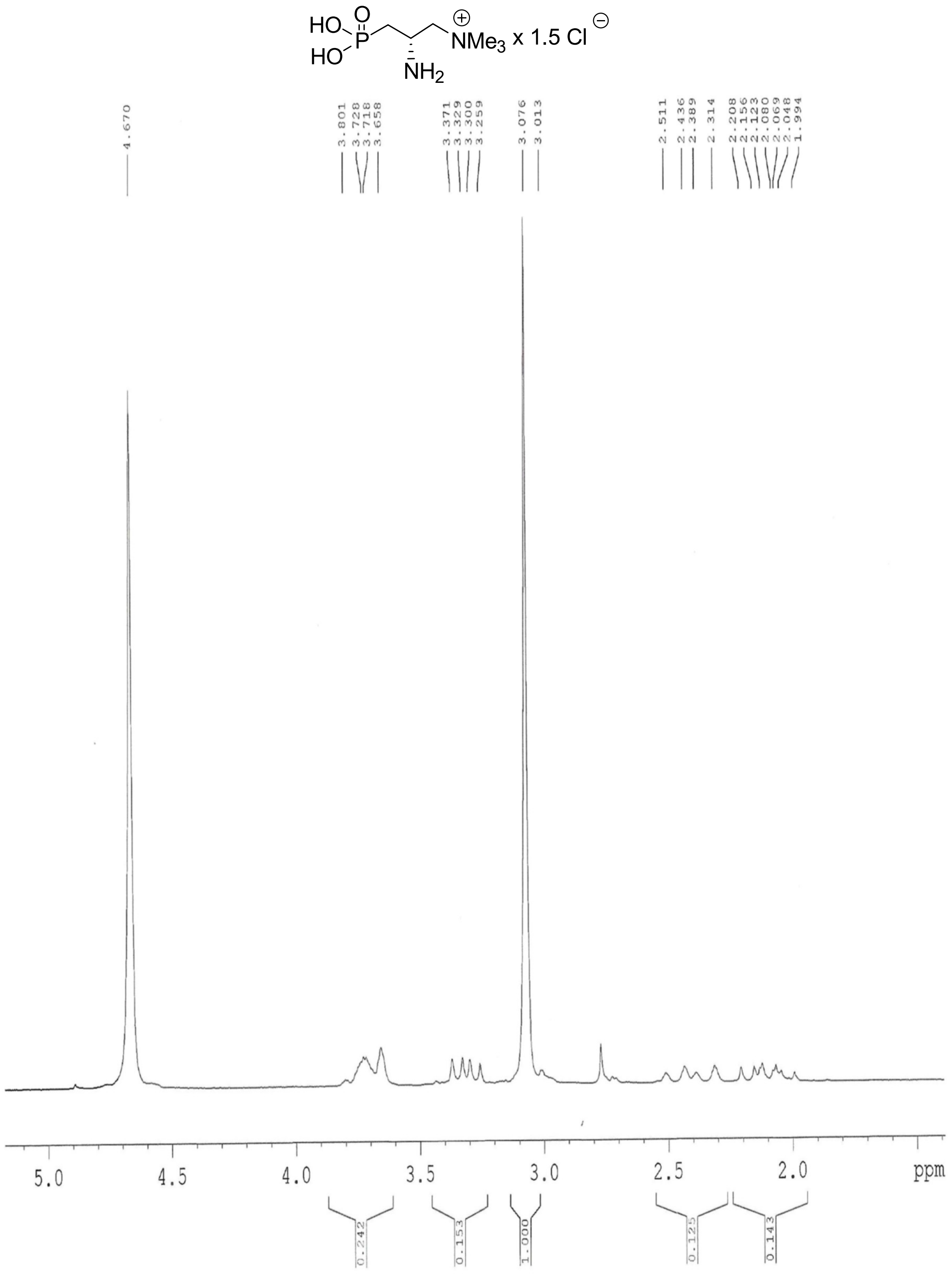

^{31}P NMR control of the progress of the enzymatic kinetic resolution of *diethyl 2-amino-3-N,N-dimethyloaminopropylphosphonate* **12**

after 2 h

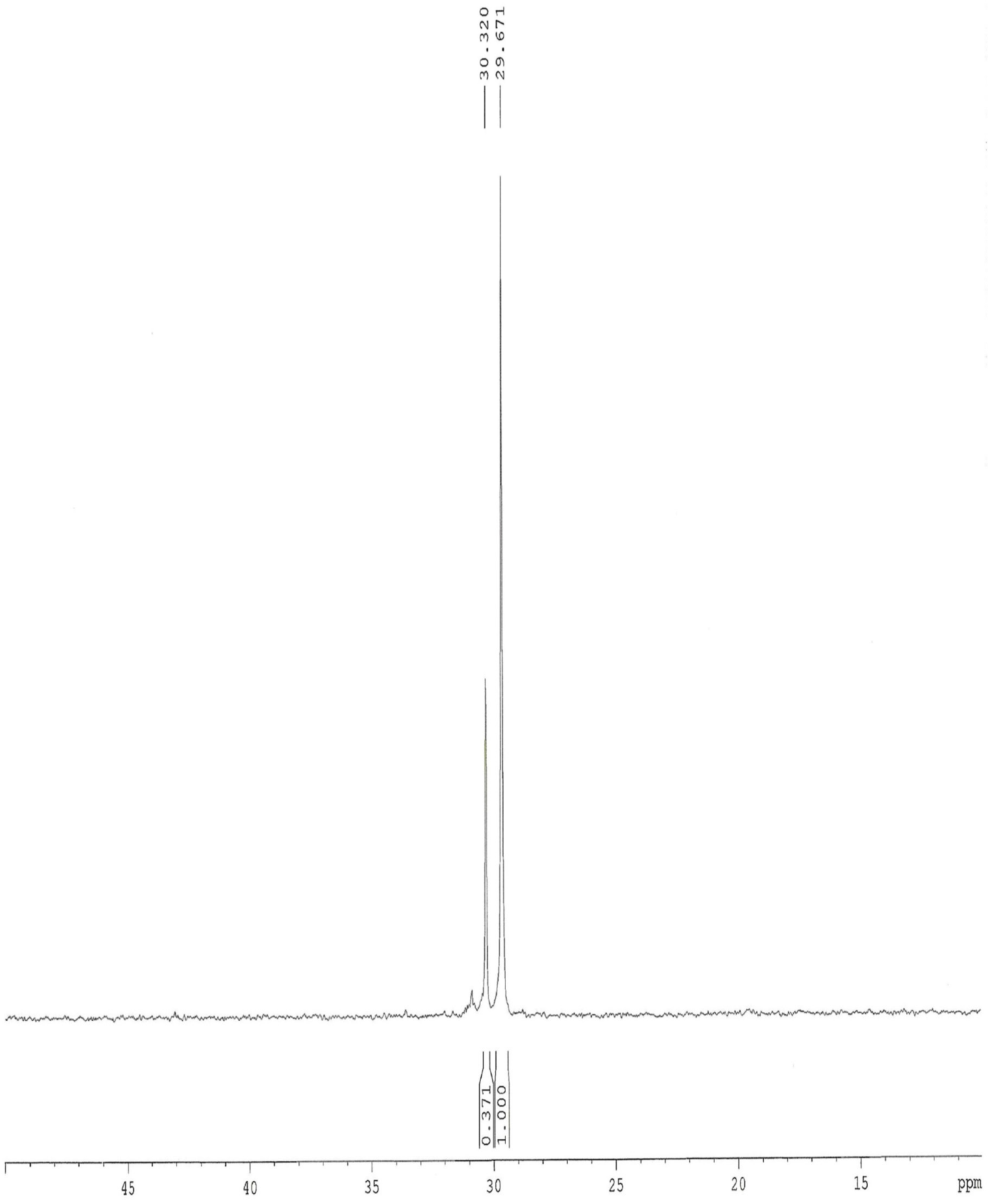

^{31}P NMR control of the progress of the enzymatic kinetic resolution of *diethyl 2-amino-3-N,N-dimethyloaminopropylphosphonate* **12**

after 4 h

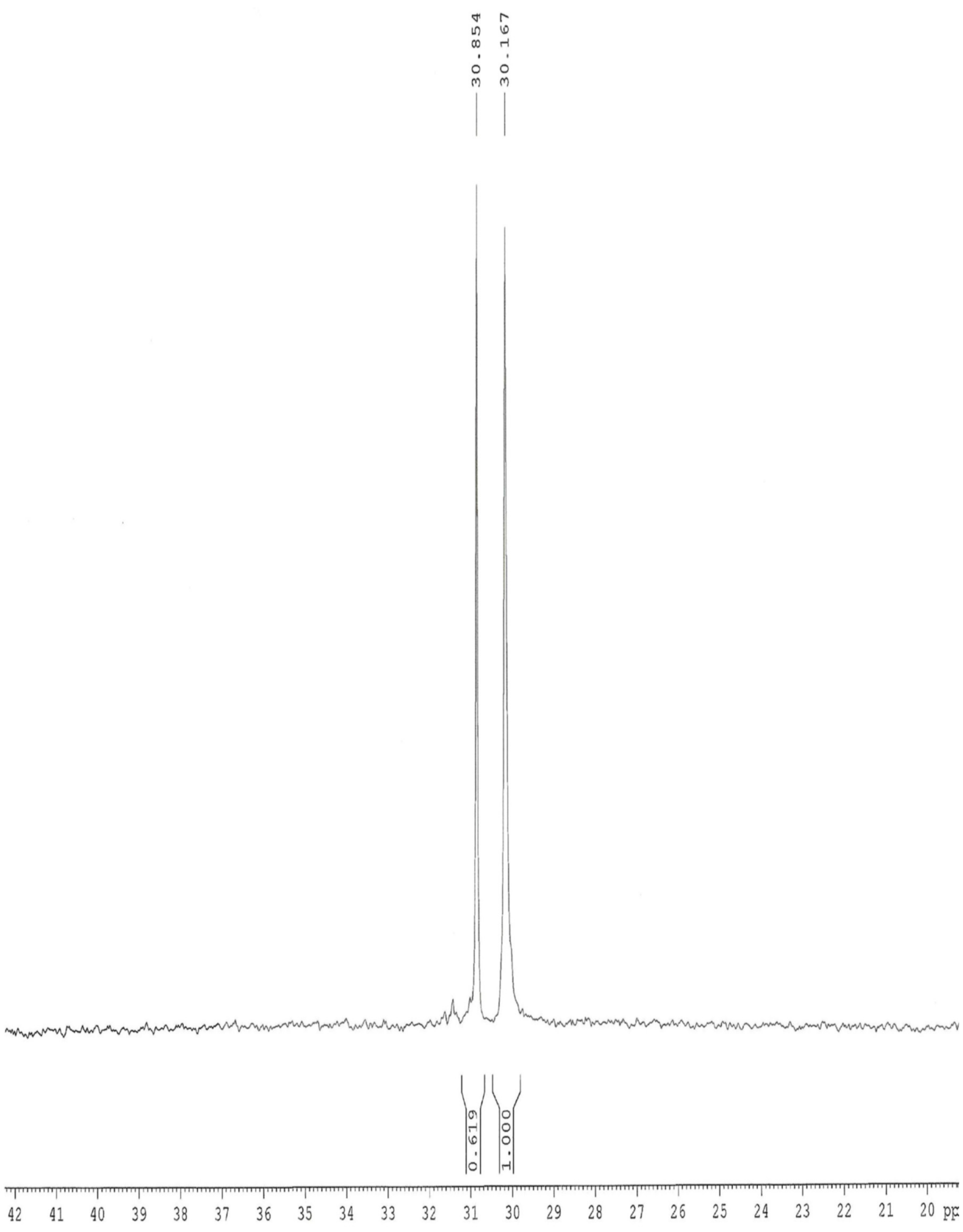

^{31}P NMR control of the progress of the enzymatic kinetic resolution of *diethyl 2-amino-3-N,N-dimethyloaminopropylphosphonate* **12**

after 7 h

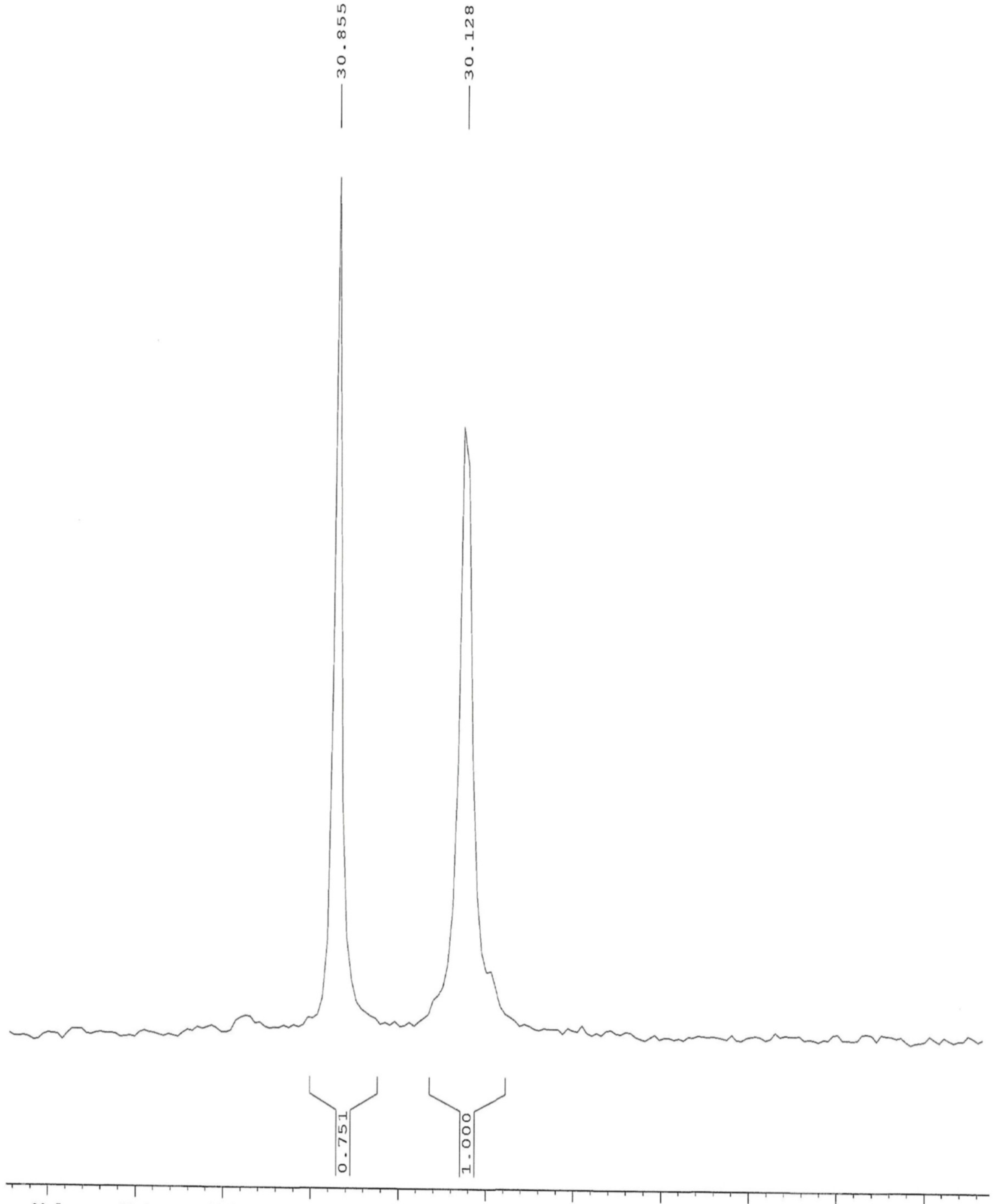

^{31}P NMR control of the progress of the enzymatic kinetic resolution of *diethyl 2-amino-3-N,N-dimethyloaminopropylphosphonate* **12**

after 24 h

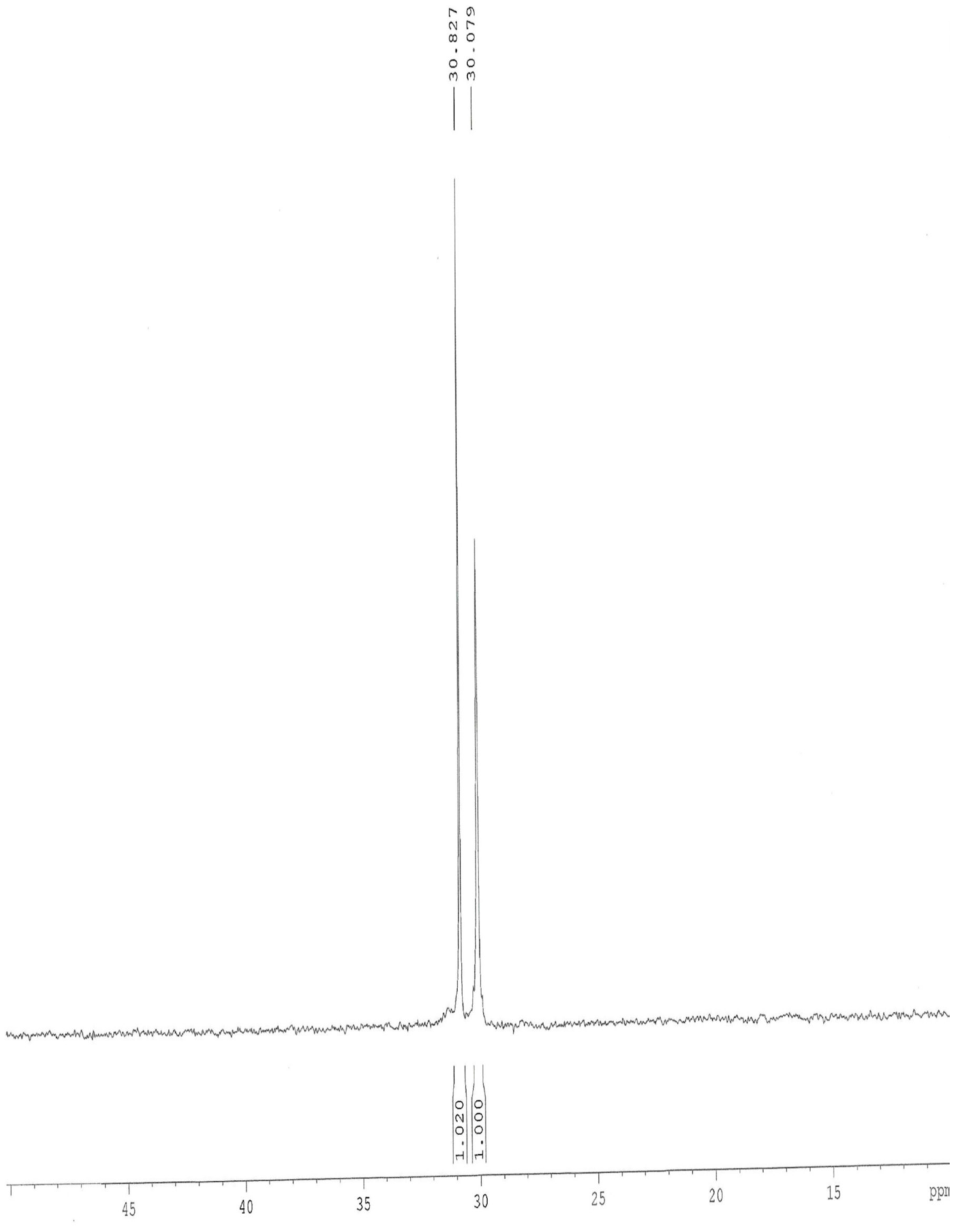

Determination of enantiomeric excess of *diethyl 2-amino-3-N,N-dimethyloaminopropylphosphonate* **12**

^{31}P NMR ($CDCl_3$) of racemic **12** with (+)-(*R*)-*t*-butylphenylphosphinothioic acid

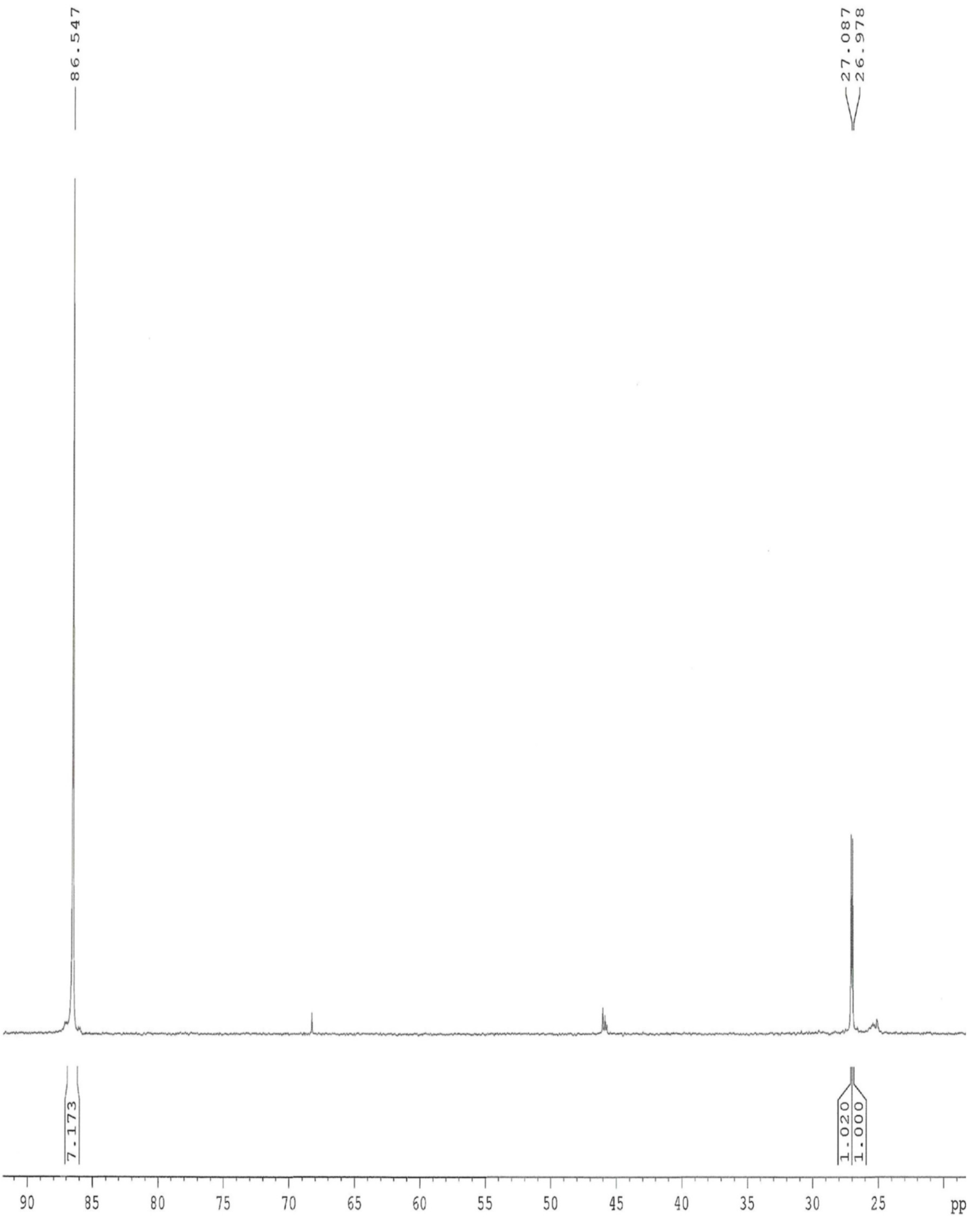

Determination of enantiomeric excess of *diethyl 2-amino-3-N,N-*

dimethyloaminopropylphosphonate **12**
^{31}P NMR (CDCl$_3$) of racemic **12** with (+)-(*R*)-*t*-butylphenylphosphinothioic acid; extension of the amine signals

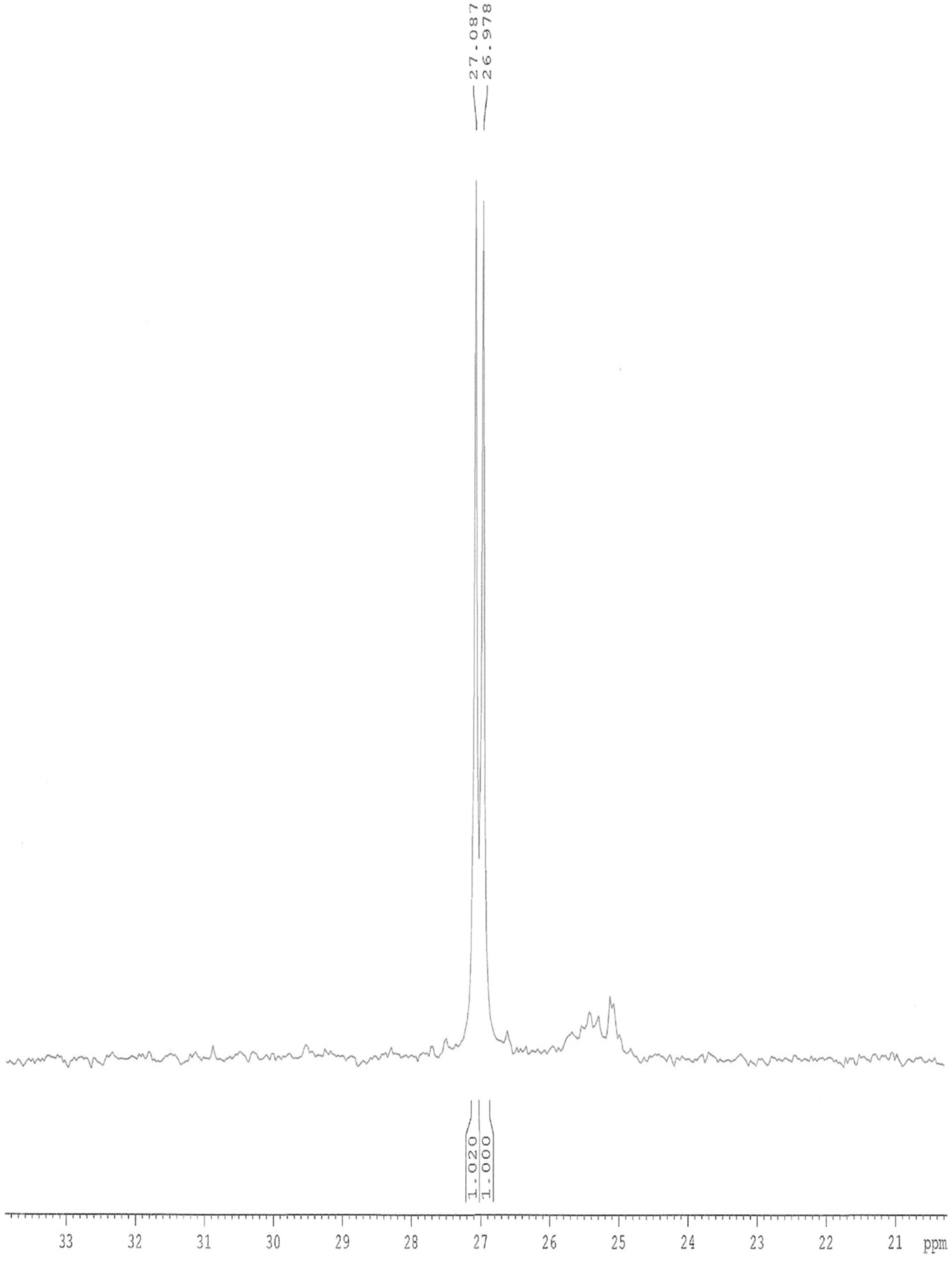

Determination of enantiomeric excess of *diethyl 2-amino-3-N,N-*

dimethyloaminopropylphosphonate **12**
^{31}P NMR ($CDCl_3$) of enantiomerically enriched (+)-(*R*)-**12**
with (+)-(*R*)-*t*-butylphenylphosphinothioic acid

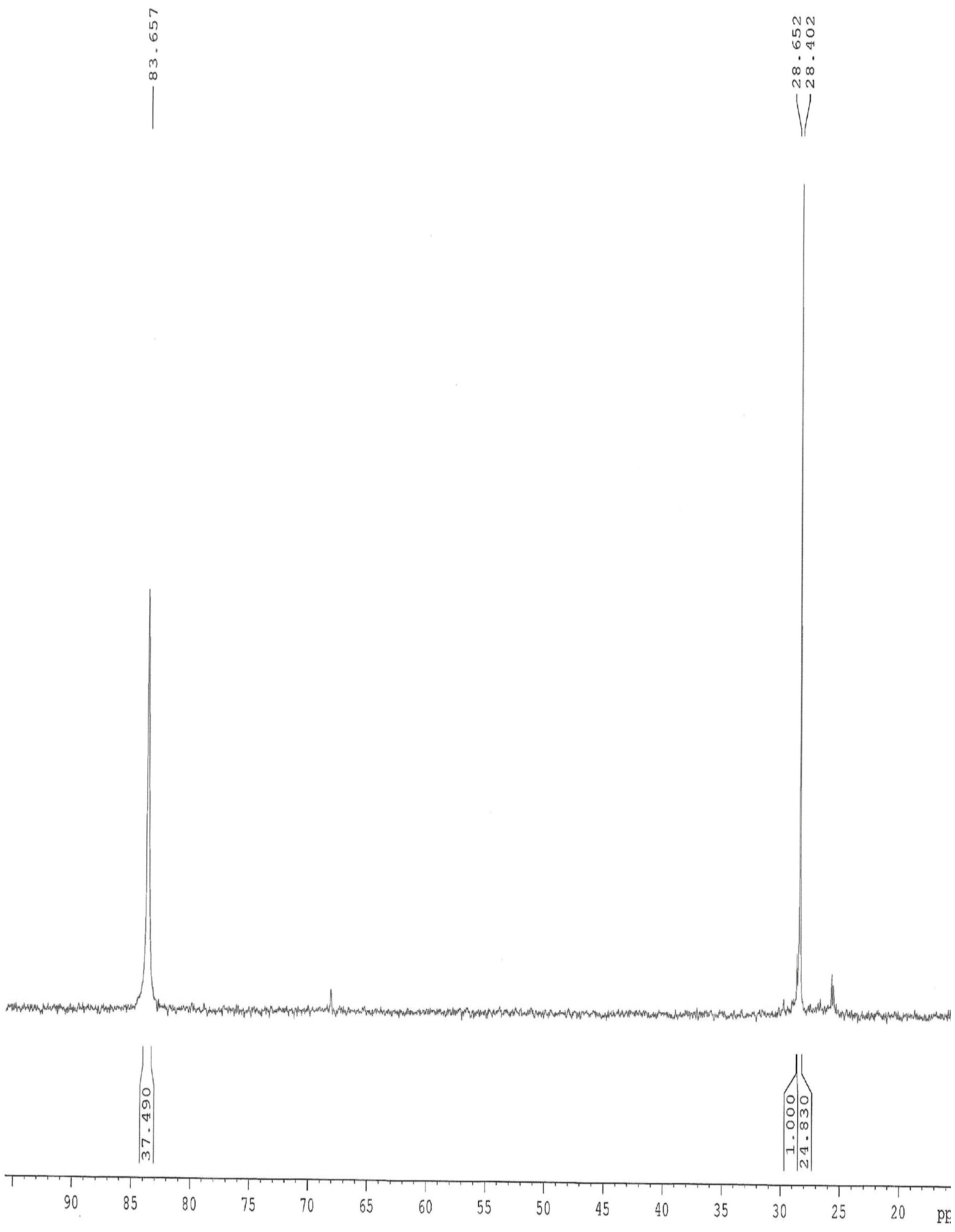

Determination of enantiomeric excess of *diethyl 2-amino-3-N,N-dimethyloaminopropylphosphonate* **12**
^{31}P NMR ($CDCl_3$) of enantiomerically enriched (+)-(*R*)-**12**
with (+)-(*R*)-*t*-butylphenylphosphinothioic acid

extension of the amine signals

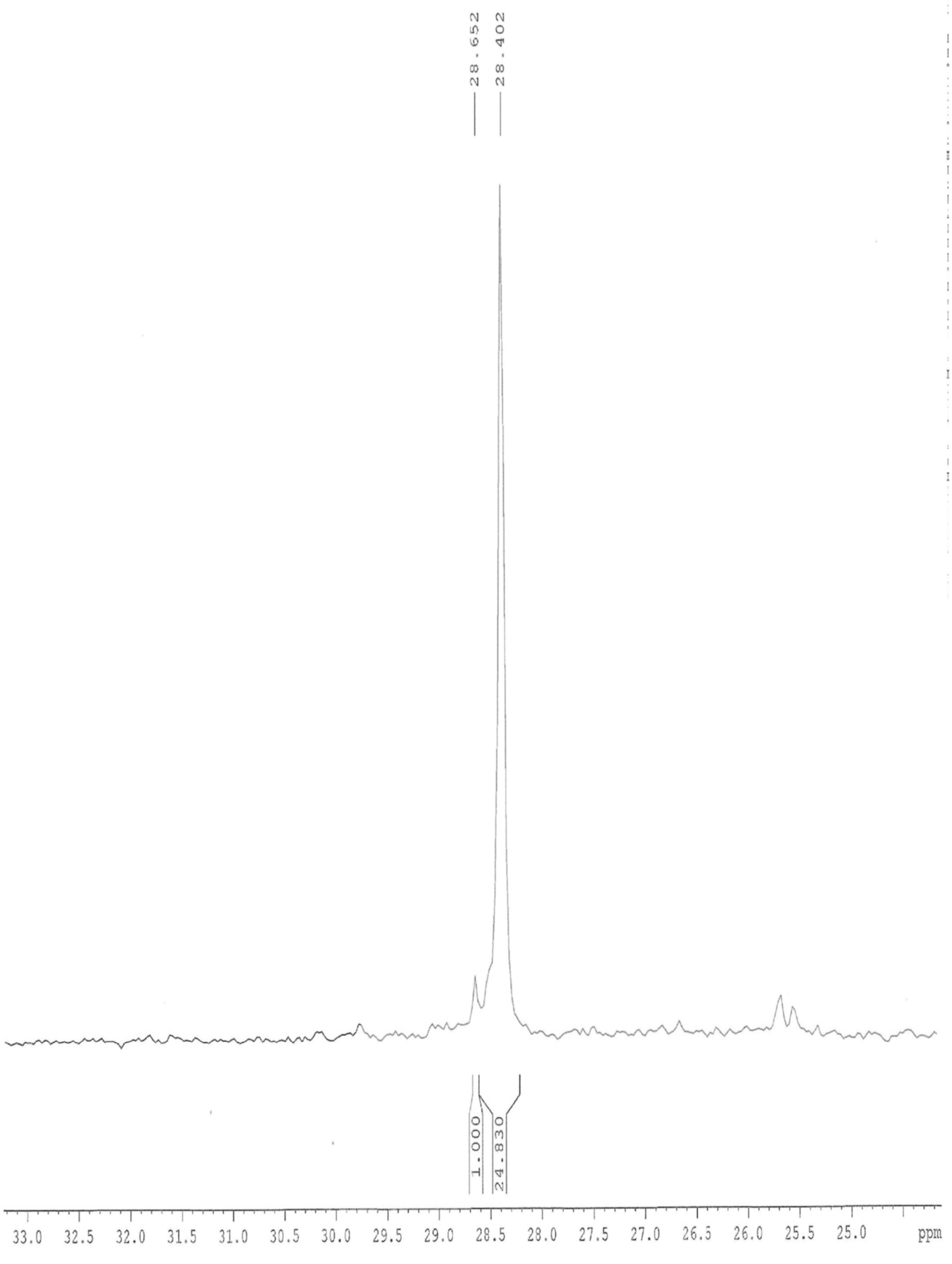

Supplement: Supplementary file 1 [file molecules-29-01799-s001.zip › molecules-2944829-supplementary.pdf]
